# In vivo detection of dysregulated choline metabolism in paclitaxel-resistant ovarian cancers with proton magnetic resonance spectroscopy

**DOI:** 10.1186/s12967-022-03292-z

**Published:** 2022-02-15

**Authors:** Jing Lu, Ying Li, Yong Ai Li, Li Wang, An Rong Zeng, Xiao Liang Ma, Jin Wei Qiang

**Affiliations:** 1 Department of Radiology, Jinshan Hospital, Fudan University, 1508 Longhang Road, Shanghai, 201508 People’s Republic of China; 2 Department of Pathology, Jinshan Hospital, Fudan University, 1508 Longhang Road, Shanghai, 201508 People’s Republic of China

**Keywords:** Ovarian cancer, Drug resistance, Proton magnetic resonance spectroscopy, Metabolomics, Choline metabolism

## Abstract

**Background:**

Chemoresistance gradually develops during treatment of epithelial ovarian cancer (EOC). Metabolic alterations, especially in vivo easily detectable metabolites in paclitaxel (PTX)-resistant EOC remain unclear.

**Methods:**

Xenograft models of the PTX-sensitive and PTX-resistant EOCs were built. Using a combination of in vivo proton-magnetic resonance spectroscopy (^1^H-MRS), metabolomics and proteomics, we investigated the in vivo metabolites and dysregulated metabolic pathways in the PTX-resistant EOC. Furthermore, we analyzed the RNA expression to validate the key enzymes in the dysregulated metabolic pathway.

**Results:**

On in vivo ^1^H-MRS, the ratio of (glycerophosphocholine + phosphocholine) to (creatine + phosphocreatine) ((GPC + PC) to (Cr + PCr))(i.e. Cho/Cr) in the PTX-resistant tumors (1.64 [0.69, 4.18]) was significantly higher than that in the PTX-sensitive tumors (0.33 [0.10, 1.13]) (*P* = 0.04). Forty-five ex vivo metabolites were identified to be significantly different between the PTX-sensitive and PTX-resistant tumors, with the majority involved of lipids and lipid-like molecules. Spearman’s correlation coefficient analysis indicated in vivo and ex vivo metabolic characteristics were highly consistent, exhibiting the highest positive correlation between in vivo GPC + PC and ex vivo GPC (r = 0.885, *P* < 0.001). These metabolic data suggested that abnormal choline concentrations were the results from the dysregulated glycerophospholipid metabolism, especially choline metabolism. The proteomics data indicated that the expressions of key enzymes glycerophosphocholine phosphodiesterase 1 (GPCPD1) and glycerophosphodiester phosphodiesterase 1 (GDE1) were significantly lower in the PTX-resistant tumors compared to the PTX-sensitive tumors (both *P* < 0.01). Decreased expressions of GPCPD1 and GDE1 in choline metabolism led to an increased GPC levels in the PTX-resistant EOCs, which was observed as an elevated total choline (tCho) on in vivo ^1^H-MRS.

**Conclusions:**

These findings suggested that dysregulated choline metabolism was associated with PTX-resistance in EOCs and the elevated tCho on in vivo ^1^H-MRS could be as an indicator for the PTX-resistance in EOCs.

**Supplementary Information:**

The online version contains supplementary material available at 10.1186/s12967-022-03292-z.

## Background

Epithelial ovarian cancer (EOC) is the most lethal gynecological malignancy and is the seventh most commonly diagnosed cancer among women worldwide, with 75% of patients diagnosed in the advanced stage and 46% exhibiting a 5-year survival rate after diagnosis [1]. The current standard treatment approach comprises cytoreductive surgery followed by combined chemotherapy with platinum and paclitaxel (PTX) [2]. The majority of the patients have an initial response to chemotherapy, e.g., cisplatin and PTX [3]. However, PTX resistance gradually develops during treatment, leading to therapeutic failure [4]. More than 75% of patients are likely to suffer tumor recurrence due to chemoresistance [2].

Metabolomics analysis can provide a real-world assessment of cancer cell physiology [5, 6]. A close relationship between deregulated metabolic reprogramming and drug resistance in cancer therapy has been reported [7–10]. Targeting metabolism might represent a potential option to overcome drug resistance [11]. Therefore, a better understanding of the adaptive tumor phenotype following treatment resistance and exploring the clinically applicable biomarkers of chemoresistance, are required to retrieve the chemosensitivity in EOC patients [3, 11]. However, metabolic alterations, especially easily detectable metabolites, in PTX-resistant EOC remain unclear.

Metabolic changes have most commonly been studied using magnetic resonance spectroscopy (MRS) [12], which is a non-invasive functional imaging method on magnetic resonance imaging (MRI). A great advantage of MRS is the ability to detect intrinsic metabolic changes without the administration of an extrinsic marker [13]. Compared to benign ovarian tumors, an increased total choline (tCho) on proton (^1^H)-MRS has been considered to be a characteristic manifestation of EOC [14–17]. ^1^H-MRS studies report alterations in the spectral profile in the region of 3.20 to 3.24 ppm, which are indicative of tCho levels including glycerophosphocholine (GPC), phosphocholine (PC), and free Cho [12]. Moreover, metabolic changes have been reported after chemotherapy in malignant tumors, such as breast cancer and glioma, suggesting that these metabolic changes can be used as indicators of therapeutic response [18–22]. However, ^1^H-MRS studies concerning chemoresistance in EOC have not been performed.

**Fig. 1:**
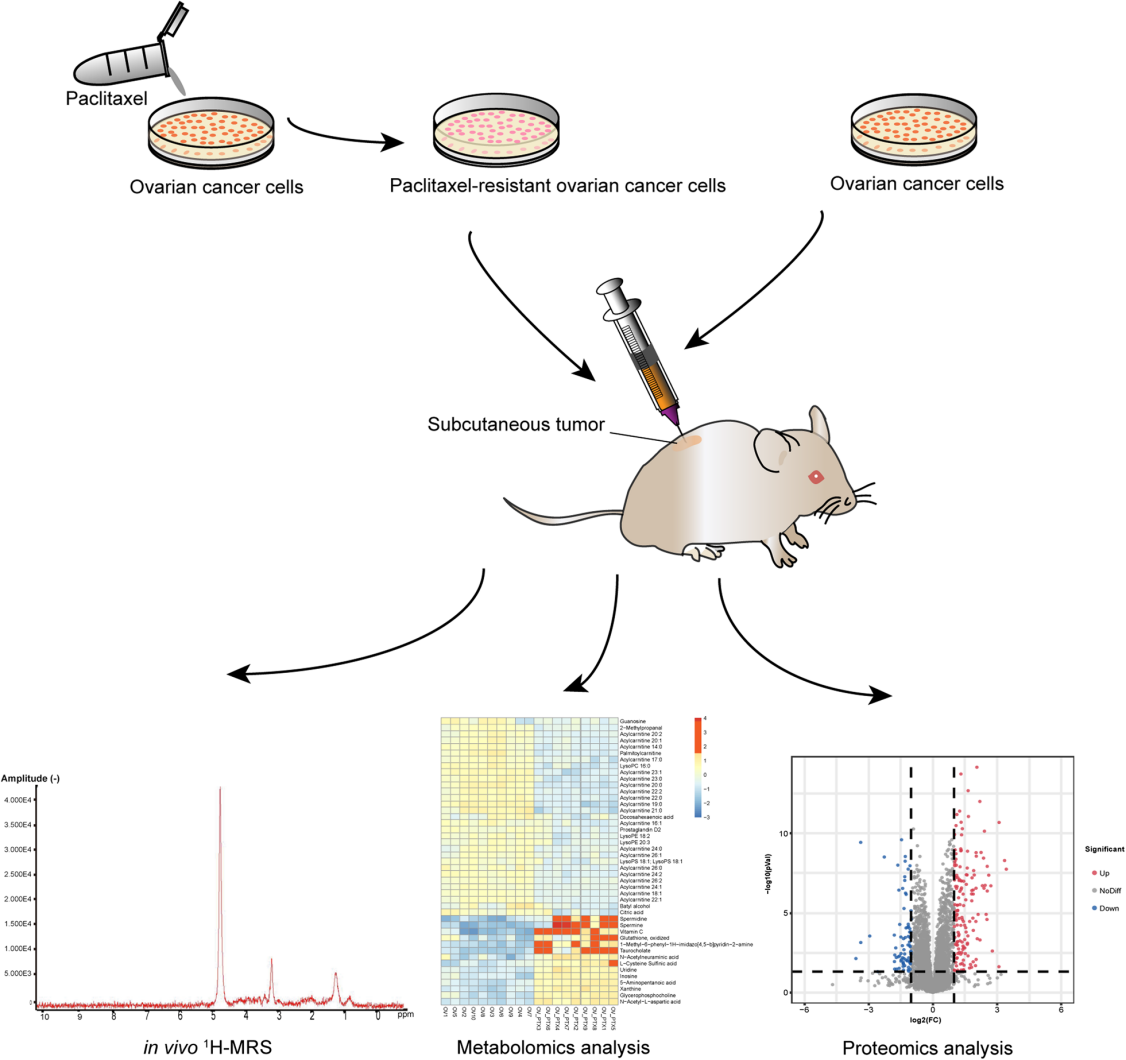
The flow chart of the research process. (^1^H-MRS: proton-magnetic resonance spectroscopy)

In this study, we used a combination of in vivo ^1^H-MRS, metabolomics and proteomics to perform an unbiased characterisation of tumors from PTX-resistant xenograft models (Fig. 1). We show that abnormal choline metabolism leads to PTX resistance in EOC and this can be accurately demonstrated by in vivo ^1^H-MRS. In addition, we identify glycerophosphocholine phosphodiesterase 1 (GPCPD1) and glycerophosphodiester phosphodiesterase 1 (GDE1) as key enzymes associated with PTX resistance, representing a potential therapeutic target.

## Methods

### Cell culture

All cells were cultured in RPMI 1640 medium (Sigma Aldrich, St Louis, MI, USA) with 10% fetal bovine serum (FBS, Gibco, Thermo Fisher Scientific, Waltham, MA, USA) and maintained at 37 °C under 5% CO_2_. PTX-resistant OVCAR-3 (OV_PTX) cells were generated in the laboratory and kindly provided by Dr. GX Xu (Fudan University, China) [23]. OV_PTX cells were derived from parental OVCAR-3 (OV) cells by treating cells with the PTX (Sichuan Taiji Pharm, China) regimen through a gradually increasing PTX dose in RPMI 1640 medium with 10% FBS.

### Proliferation assay

Cell proliferation was measured by Cell Counting Kit-8 (CCK-8, Dojindo Molecular Technologies Inc., Shanghai, China). OV and OV_PTX cells were plated in 96-well plates with a density of 5 × 10^3^ cells per well and incubated for 24 h. PTX was added with increasing concentrations from 0.001–10 μmol/mL to cells, which were then incubated for 48 h. The cells were then incubated with 10 μL of CCK-8 per well for 1 h at 37 °C. Absorbance was measured at a wavelength of 450 nm using a Bio-Tek ELX808IU absorbance microplate reader (Bio-Tek Instruments Inc., USA).

### Xenograft models

Female BALB/c nude mice (Jiesijie Laboratory Animal Company, Shanghai, China; age, 4–5 weeks; weight, 12–15 g) were used under approved animal care. In vivo experiments were performed in accordance with the guidelines formulated by the Ethics Review Committee of China Animal Experimental System and were approved by the ethics committee of Shanghai Municipal Public Health Clinical Center (No. 2020-A019-01). Twenty nude mice were randomly divided into the OV group and OV_PTX group (n = 10 in each group). Briefly, 5 × 10^6^ OV or OV_PTX cells/mouse were suspended in serum-free medium, and 0.1 mL of the cell suspension was injected subcutaneously in the right anterior limb. Mice were monitored daily and weighted every two days. Tumor size was measured with a caliper every two days for the greatest longitudinal diameter (length) and greatest transverse diameter (width). Volume was calculated using the modified elliptical formula (length × width^2^)/2 [24]. Tumors were allowed to grow for 19 days after injection until a diameter of approximately 1.0 cm was measured, which were suitable for in vivo ^1^H-MRS. One mouse in the OV_PTX group died before imaging.

### In vivo ^1^H-MRS

Mice with OV or OV_PTX tumors were anesthetized with isoflurane (1.5–2%) in oxygen (1 L/min) and imaged in the prone position in a 7.0 T Biospec small-animal MRI scanner (Bruker Corporation, Billerica, MA, USA). The imaging protocol included the following sequences:

1. T2-weighted rapid acquisition relaxation enhance: time of echo, 35 ms; time of repetition, 2500 ms; slice thickness, 0.4 mm; field of view, 20 × 20 mm; matrix, 256 × 256; and number of averaged scans, 8.
2. Single voxel point-resolved spectroscopy ^1^H-MRS: time of echo, 16 ms; time of repetition, 2500 ms; voxel size, 1.5 × 1.5 × 1.5 mm; number of averaged scans, 128; and scan time, 5 min 20 s.

The tumors were scanned on the transverse, coronal and sagittal planes using the T2 rapid acquisition relaxation enhance sequence for the three-dimensional positioning of ^1^H-MRS. Metabolite spectral fitting was performed using LCModel Version 6.3-0I, with a basis set provided by the LCModel software for a 7.0 T Bruker MRI scanner with time of echo = 16 ms [25]. Relative metabolite concentrations and their uncertainties were estimated by fitting the spectrum to a linear combination of basis spectra of each individual metabolite. The unsuppressed water spectrum was used to normalize the initial fit to generate a first estimate of metabolite concentration by scaling the relative areas and chemical shifts across the two sets of spectra. The spectral range for the analysis was set to 0.2–4.0 ppm to contain most peaks of interest: alanine (Ala), aspartate (Asp), creatine (Cr), phosphocreatine (PCr), glycerophosphocholine (GPC), phosphocholine (PC), inositol (Ins), lactate (Lac), taurine (Tau), N-acetylaspartate (NAA), N-acetylaspartylglutamate (NAAG), macromolecules (MM) 09, lipid (Lip) 09, Lip 13a, Lip 13b, MM12, MM14, MM20, Lip 20. The numbers after MM and Lip indicated the approximate chemical shift in ppm of the peaks; e.g., MM 14 for the macromolecule peak near 1.4 ppm. Only metabolite concentrations quantified with Cramèr-Rao lower bounds below 20% on average were included in further analysis [26]. With software (Fire Voxel, CAI2R, New York University, NY, USA), the region of interest was manually delineated slice-by-slice along the contour of the tumor on the transverse T2-weighted images (T2WIs) (M.X.L. and L.J., with 5 and 7 years of experience in gynecological imaging, respectively). Then, the volume of interest was postprocessed automatically for tumor anatomic measurement.

### Sample collection and histopathology

In order to acquire a true map of in vivo metabolism, tumors were collected as soon as possible after MRI scanning with mice euthanized by excess CO_2_ exposure. Tumor samples were divided into multiple parts. One part was fixed with 4% paraformaldehyde and then embedded in paraffin and stained using hematoxylin and eosin for histological feature analysis. The other parts of tumor samples were flash-frozen in liquid nitrogen and were used for metabolomics analysis, proteomics analysis and quantitative RT-PCR, respectively.

### Metabolomics analysis

Tumor samples (100 mg) were extracted for analysis using by liquid chromatography-mass spectrometry (LC–MS). Pooled quality control samples were also prepared by combining the same volume of each sample and repeatedly injected during the assay to monitor instrumental stability and avoid systematic bias. The acquired LC–MS data was pretreated using XCMS software. Features with < 50% of quality control samples or 80% of test samples were removed, and values for missing peaks were extrapolated with the k‐nearest neighbor algorithm to further improve the data quality. The group datasets were normalized before analysis. The detailed parameters were described in Additional file 1: Supplementary of metabolomics analysis method. Statistical analysis included principal component analysis (PCA), orthogonal partial least squares discriminant analysis (OPLS-DA), Student’s *t*-test and fold change analysis. The *P* value obtained by Student’s *t*‐test was then adjusted for multiple tests using a false discovery rate-reducing process (Benjamini-Hochberg) and was used to determine differential metabolites. The following criteria were used to screen the differential metabolites: variable importance in projection (VIP) sores ≥ 1, *P* value < 0.05, and fold change ≥ 2 or ≤ 0.5. Pathway enrichment analysis of differential metabolites was performed using the Kyoto Encyclopedia of Genes and Genomes and MetaboAnalyst 3.0 (Montre al, QC, Canada) databases. Bioinformatic analysis was performed using the OmicStudio tools at https://www.omicstudio.cn/tool.

### Proteomics analysis

Samples were lysed in sodium dodecyl sulfate buffer and homogenized. Proteins were digested overnight by trypsin (Promega, Madison, WI, USA), and the resulted peptides were collected as a filtrate. Pooled peptides from all samples were fractionated by reversed-phase chromatography using an Agilent 1260 infinity II HPLC (SCIEX, Framingham, MA, USA). The detailed parameters were described in Additional file 2: Supplementary of proteomics analysis method. Raw data of data-independent acquisition were processed and analyzed by Spectronaut 14.6 (Biognosys AG, Switzerland) with default settings. Data extraction was determined by Spectronaut X based on extensive mass calibration. Precursors which passed the filters were used for quantification.The average top 3 filtered peptides that passed the 1% Q- value cutoff (false discovery rate) were used to calculate the major group quantities. Significantly enriched proteins were selected using Student’s *t*-test.

### Quantitative RT-PCR

Total RNA was extracted from tumors using Trizol reagent and reverse-transcribed into cDNA with 500 ng of total RNA using the PrimeScript RT reagent kit (Thermo Fisher Scientific, Waltham, MA, USA) according to the manufacturer’s instructions. Gene expression assays were performed on an ABI StepOnePlus Real-Time PCR instrument (Thermo Fisher Scientific, Waltham, MA, USA). The following custom designed primers were purchased from GENEWIZ Inc. (Suzhou, China): GPCPD1, GTTTTTGCGATATGTGGAAGCTG (forward) and AGCGATACTGAACTGATACTCCT (reverse); GDE1, GACTGGGCGATTGTGTGATTT (forward) and AGGGTAGGGATCTTTTCATCAGG (reverse); and β-Actin, GCCGTGGTGGTGAAGCTGT (forward) and ACCCACACTGTGCCCATCTA (reverse).

### Statistical analysis

Continuous variables with normal distribution were presented as the mean ± standard deviation; nonnormally distributed variables were presented as the median (interquartile range). All statistical analyses were performed with SPSS (version 23.0, SPSS, Inc., Chicago, IL, USA). The data were analyzed using Student’s *t*-test or Mann–Whitney U test. Receiver operating characteristic curve analysis (MedCalc Software, Mariakerke, Belgium) was used to assess the diagnostic performance and determine a cutoff value for the significant metabolites from ^1^H-MRS to differentiate the PTX-sensitive and PTX-resistant tumors. The correlation of metabolites between in vivo ^1^H-MRS and ex vivo metabolomics analysis and the correlations between protein expressions and metabolite levels were analyzed using Spearman correlation tests. Differences with a *P* < 0.05 were considered statistically significant.

## Results

### In vivo metabolic profile of PTX-resistant ovarian cancer

**Fig. 2:**
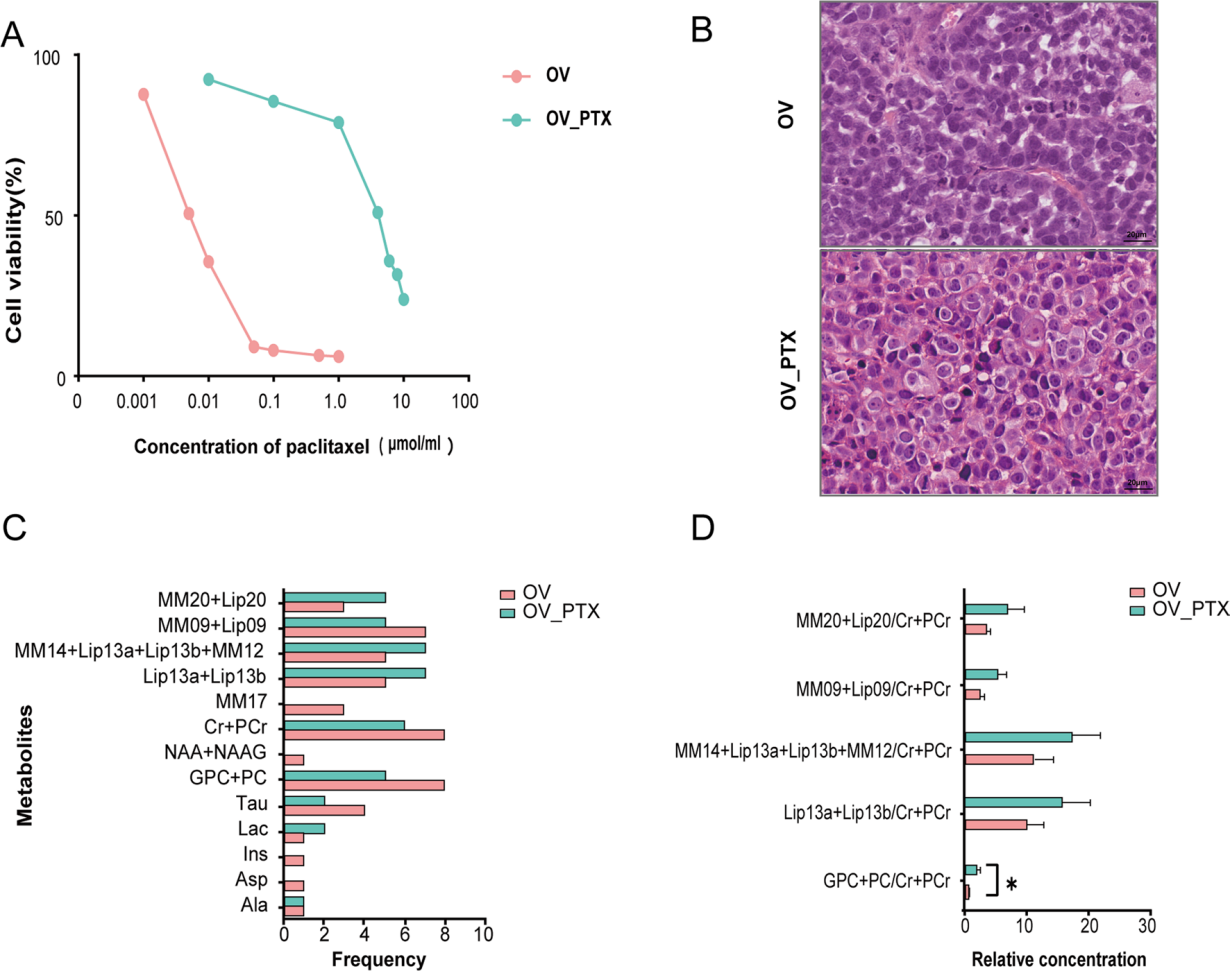
In vivo metabolic profile of the PTX-sensitive and PTX-resistant tumors. Cell proliferation of OV and OV_PTX cells treated for 48 h with increasing concentrations of PTX (**A**). Hematoxylin and eosin staining of subcutaneous tumors showing the more obvious cell atypia and nuclear mitosis in the OV_PTX tumor cells (400 × magnification, scale bars = 20 μm) (**B**). The frequencies and relative concentrations of metabolite peaks in the OV and OV_PTX groups on in vivo ^1^H-MRS (**C**, **D**). *, *P* < 0.05. *PTX* paclitaxel, *OV*: OVCAR-3

We performed the cell proliferation assay to confirm the PTX resistance, with a resistance index of 484.3 ± 166.8 (Fig. 2A). Cell atypia was more obvious in the OV_PTX tumor than that in OV tumor (Fig. 2B). Xenograft tumors were significantly larger in the OV group (1028.5 ± 370.5 mm^3^) than in the OV_PTX group (337.2 ± 224.0 mm^3^) (*P* < 0.001).

**Fig. 3:**
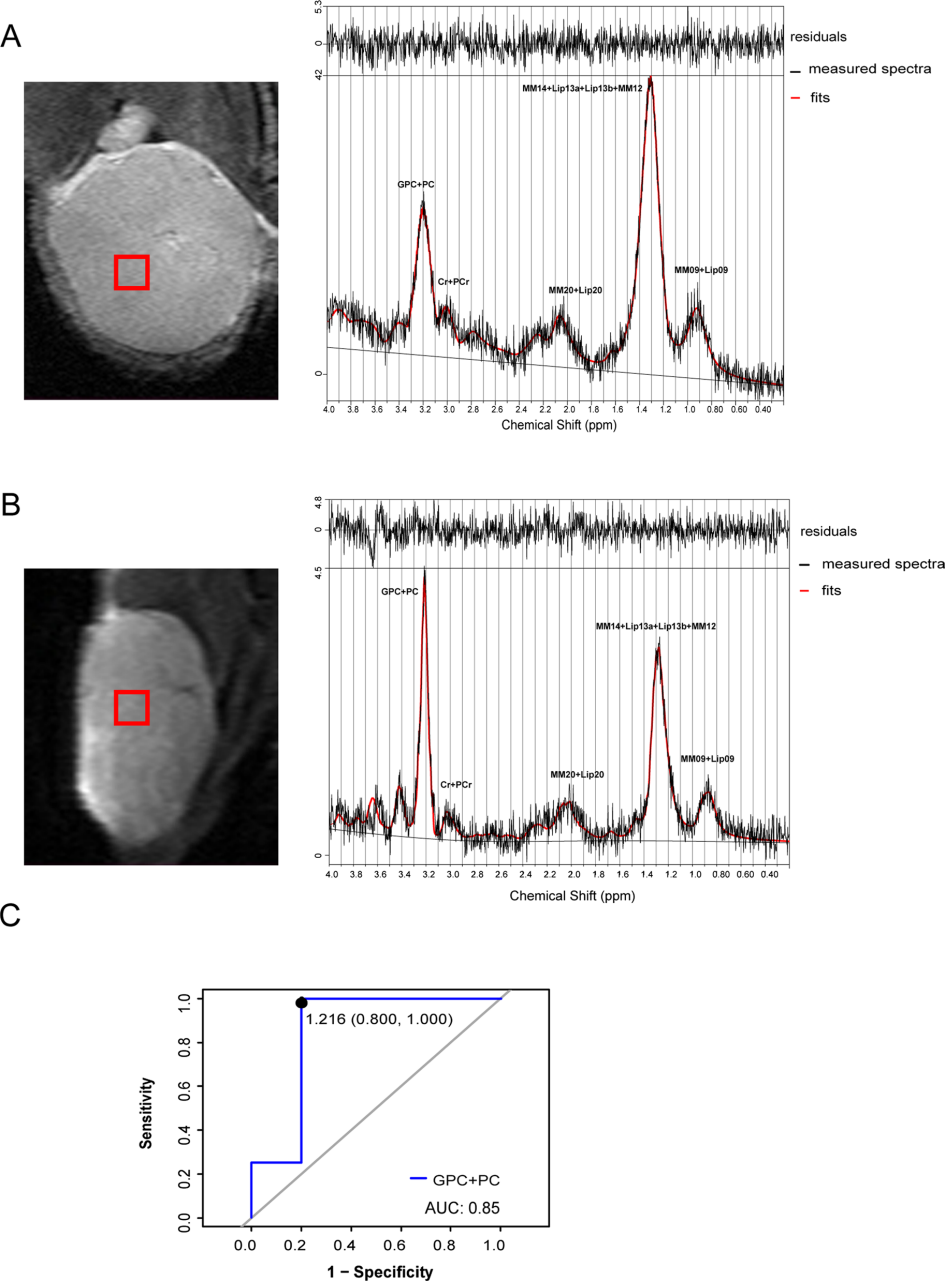
Samples of MR spectra and the diagnostic efficiency of in vivo ^1^H-MRS. ^1^H-MRS localization of the volume of interest and spectra acquired from the OV tumor (**A**) and OV_PTX tumor (**B**), along with fits and residuals of the fits resulting from LCModel quantification: Cr + PCr (3.03 ppm), GPC + PC (3.21 ppm), MM14 + Lip 13a + Lip 13b + MM12 (1.2–1.4 ppm), MM 09 + Lip 09 (0.9 ppm), MM 20 + Lip 20 (2.0 ppm). The (GPC + PC)/(Cr + PCr) ratio was 1.18 for the OV tumor and 5.45 for the OV_PTX tumor. Receiver operating characteristic curve analysis of in vivo differential metabolite (GPC + PC) for differentiating the OV from OV_PTX groups (**C**). *GPC* glycerophosphocholine, *PC* phosphocholine, *Cr* creatine, *PCr* phosphocreatine, *Lip* lipids, *MM* macromolecules

Eight mice from each group were successfully imaged. A total of 13 metabolites (Cramèr-Rao lower bounds < 20%) were analysed, but no significant differences in frequencies of metabolite peaks were observed between the OV and OV_PTX tumors (Table 1; Fig. 2C). Thereinto, PC was very difficult to resolve from GPC, as was PCr from Cr. The sum of GPC + PC was much more accurate than the individual concentrations. Usually, the units of the absolute concentrations are unknown, and only concentration ratios are meaningful. LCModel metabolite ratios relative to Cr + PCr were used for our analysis (as outlined in detail in the LCModel manual). The metabolite ratios and their differential levels in the OV and OV_PTX tumors are shown in Table 1 and Fig. 2D. The ratio of (GPC + PC) to (Cr + PCr) (i.e. Cho/Cr) in the OV_PTX group (1.64 [0.69, 4.18]) was significantly higher than that in the OV group (0.33 [0.10, 1.13]) (*P* = 0.04). The metabolites of interest are displayed in Fig. 3A and B. For differentiating the two groups of tumors, the optimal cutoff value of the (GPC + PC)/(Cr + PCr) ratio was 1.216, which yielded the sensitivity, specificity, and area under the curve (AUC) values of 100%, 80%, and 0.85 (95% confidence interval, 0.550–0.982), respectively (Fig. 3C). No significant differences in other 4 metabolite ratios were found between the OV and OV_PTX tumors, although higher ratios were detected in the OV_PTX tumors.

**Table 1:** Metabolites detected in the OV and OV_PTX groups using ^1^H-MRS

Metabolites and ratios	OV (n = 8)	OV_PTX (n = 8)	*P* value
Metabolite peaks^a^			
Ala	1	1	–
Asp	1	0	–
Ins	1	0	–
Lac	1	2	–
Tau	4	2	0.61
GPC + PC	8	5	0.20
NAA + NAAG	1	0	–
Cr + PCr	8	6	0.47
MM17	3	0	–
Lip13a + Lip13b	5	7	0.57
MM14 + Lip13a + Lip13b + MM12	5	7	0.57
MM09 + Lip09	7	5	0.57
MM20 + Lip20	3	5	0.62
Metabolite concentration ratios (/Cr + PCr)^b^			
GPC + PC	0.33 (0.10, 1.13)	1.64 (0.69, 4.18)	0.04
Lip13a + Lip13b	11.48 (5.88, 18.28)	15.15 (9.96, 31.86)	0.43
MM14 + Lip13a + Lip13b + MM12	12.95 (6.22, 20.42)	17.14 (12.15, 32.56)	0.43
MM09 + Lip09	3.10 (0.84, 3.58)	4.65 (3.40, 9.92)	0.09
MM20 + Lip20	3.28 (3.13, 5.56)	4.19 (2.75, 15.25)	0.46

^*1*^*H-MRS* proton-magnetic resonance spectroscopy, *OV* ovarian cancer, *OV_PTX* paclitaxel-resistant ovarian cancer, *Ala* alanine, *Asp* aspartate, *Ins* inositol, *Lac* lactate, *Tau* taurine, *GPC + PC* glycerophosphocholine + phosphocholine, *NAA + NAAG* N-acetylaspartate + N-acetylaspartatglutamate, *Cr + PCr* creatine + phosphocreatine, *Lip* lipids, *MM* macromolecules, the numbers after MM and Lip indicate the approximate chemical shift in ppm of the peaks: e.g., MM17: macromolecule peak near 1.7 ppm

^a^Chi-square test

^b^Mann–Whitney U test

### Ex vivo metabolic profile of PTX-resistant ovarian cancer

**Fig. 4:**
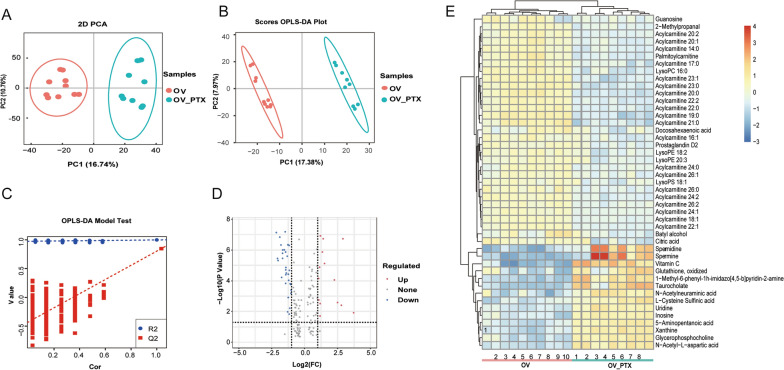
Ex vivo metabolic differences between the PTX-sensitive and PTX-resistant tumors. PCA scatter plot of the metabolite profile of positive ionization mode showing the separation between the OV and OV_PTX groups (**A**). OPLS-DA analysis showing a good discrimination between the OV and OV_PTX groups, with R2 = 0.97, Q2 = -0.46 (**B**, **C**). Data was screened using VIP ≥ 1, fold change ≥ 2 or ≤ 0.5, and P < 0.05. Volcano plot showing differential metabolites between the OV and OV_PTX groups (**D**). Hierarchical clustering heat map analysis of differential metabolites between the OV and OV_PTX groups (**E**). Each column depicts a sample and each row represents a metabolite. The colour of each section corresponds to a normalized concentration value of each metabolite. *PCA* principal component analysis, *OPLS-DA* orthogonal partial least squares discriminant analysis

**Fig. 5:**
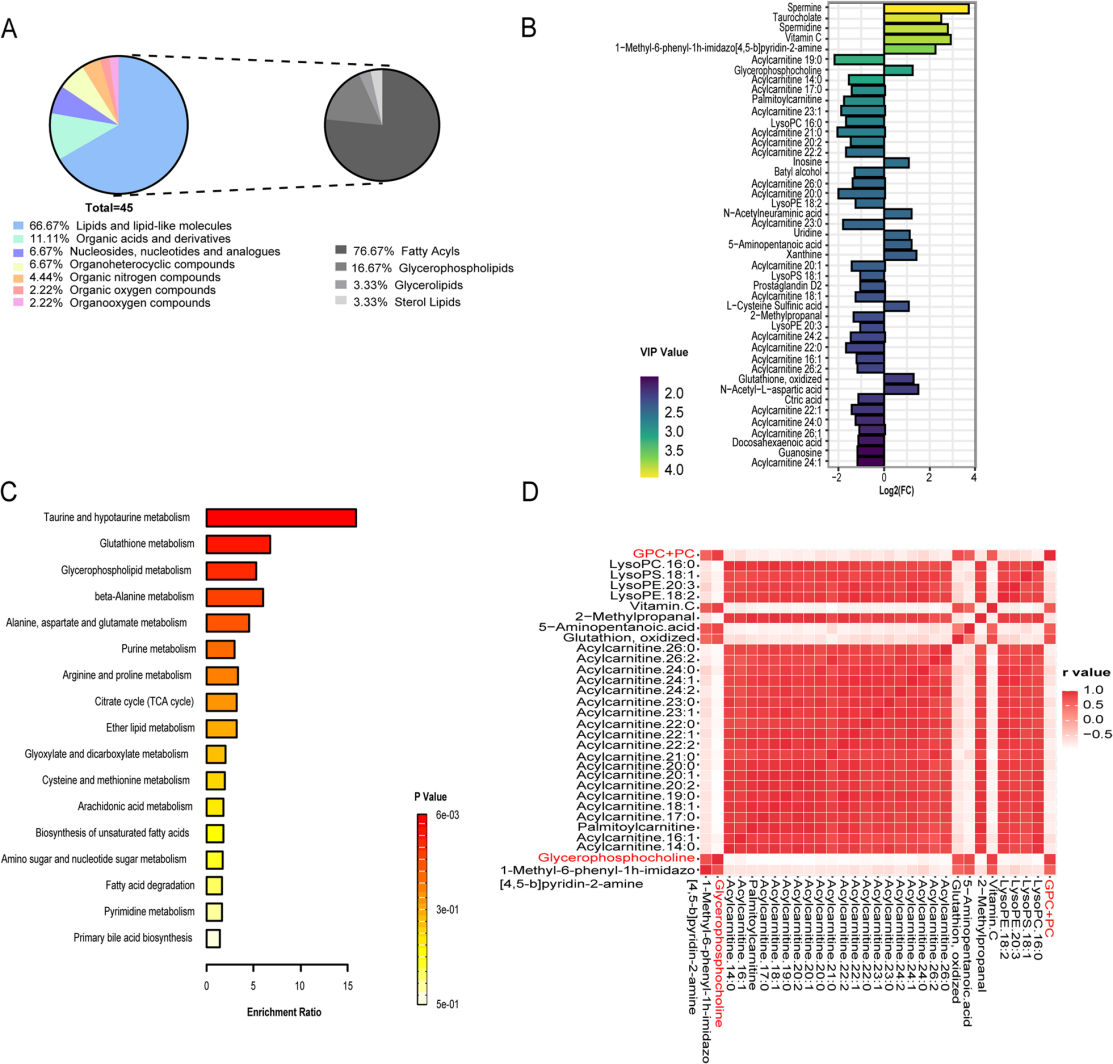
Ex vivo differential metabolites pathway analysis between the PTX-sensitive and PTX-resistant tumors and the correlation between in vivo and ex vivo differential metabolites. Composition of ex vivo differential metabolites (**A**). Linear discriminant analysis effect size of ex vivo differential metabolites (**B**). Pathway enrichment analysis of differential metabolites between the OV and OV_PTX groups (**C**). Metabolic alterations of the most relevant pathways affected by drug resistance were analyzed by MetaboAnalyst 3.0 databases. Correlation heat map of differential metabolites between in vivo and ex vivo metabolite profiles (**D**). Each square represents a metabolite and the colour corresponds to its correlation coefficient value compared with other metabolites

To decipher the mechanisms of in vivo metabolic imaging alterations associated with treatment resistance, we characterized the metabolome of the OV and OV_PTX tumors from xenograft models. As shown in the PCA plot, the metabolites of the OV group and the OV_PTX group were well separated (Fig. 4A; Additional file 3: Fig. S1A). Next, the OPLS-DA model was used to further analyze the compounds responsible for the differences between the two groups. Goodness of fit values and predictive ability values indicated that the model possessed a satisfactory fit with a good predictive power (Fig. 4B, C; Additional file 3: Fig. S1B, C). Differential metabolites were screened using VIP and fold change as thresholds. Metabolites that met VIP ≥ 1, fold change ≥ 2 or ≤ 0.5, and *P* < 0.05 were considered to be differential metabolites (Fig. 4D, E). Forty-five ex vivo metabolites were identified to be significantly different between the OV tumors and the OV_PTX tumors, with the majority involving of lipids and lipid-like molecules (66.67%, Fig. 5A, Additional file 4: Table S1). Compared to those in the OV tumors, the levels of 14 metabolites increased while 31 ones decreased in the OV_PTX tumors. Metabolomics analysis showed the top ten differential metabolites according to VIP values: spermine, taurocholate, spermidine, vitamin C, 1-methyl-6-phenyl-1H-imidazo[4,5-b]pyridin-2-amine, acylcarnitine 19:0, glycerophosphocholine, acylcarnitine 14:0, acylcarnitine 17:0, and palmitoylcarnitine (Fig. 5B). Those may be potential biomarkers to identify the PTX-resistant EOC.

Pathway enrichment analysis was performed to identify dysregulated pathways in PTX-resistant tumors. It showed that the commonly dysregulated pathways included lipid metabolism, glycometabolism, amino acid metabolism, nucleotide metabolism, and energy metabolism (Fig. 5C). The top 3 related metabolism pathways were taurine and hypotaurine metabolism, glutathione metabolism and glycerophospholipid metabolism.

The correlation between in vivo differential metabolite (GPC + PC) and every ex vivo differential metabolite was investigated by calculating the Spearman’s correlation coefficient. There were 30 ex vivo differential metabolites that correlated significantly with the differential metabolite GPC + PC (|r|> 0.5, *P* < 0.05) observed on in vivo ^1^H-MRS, exhibiting the highest positive correlation with GPC (r = 0.885, *P* < 0.001) (Fig. 5D). Overall, these data indicated that abnormal Cho concentrations were detected in the PTX-resistant EOCs because of the dysregulated glycerophospholipid metabolism, especially Cho metabolism.

### Metabolic enzyme modulation in PTX-resistant ovarian cancer

**Fig. 6:**
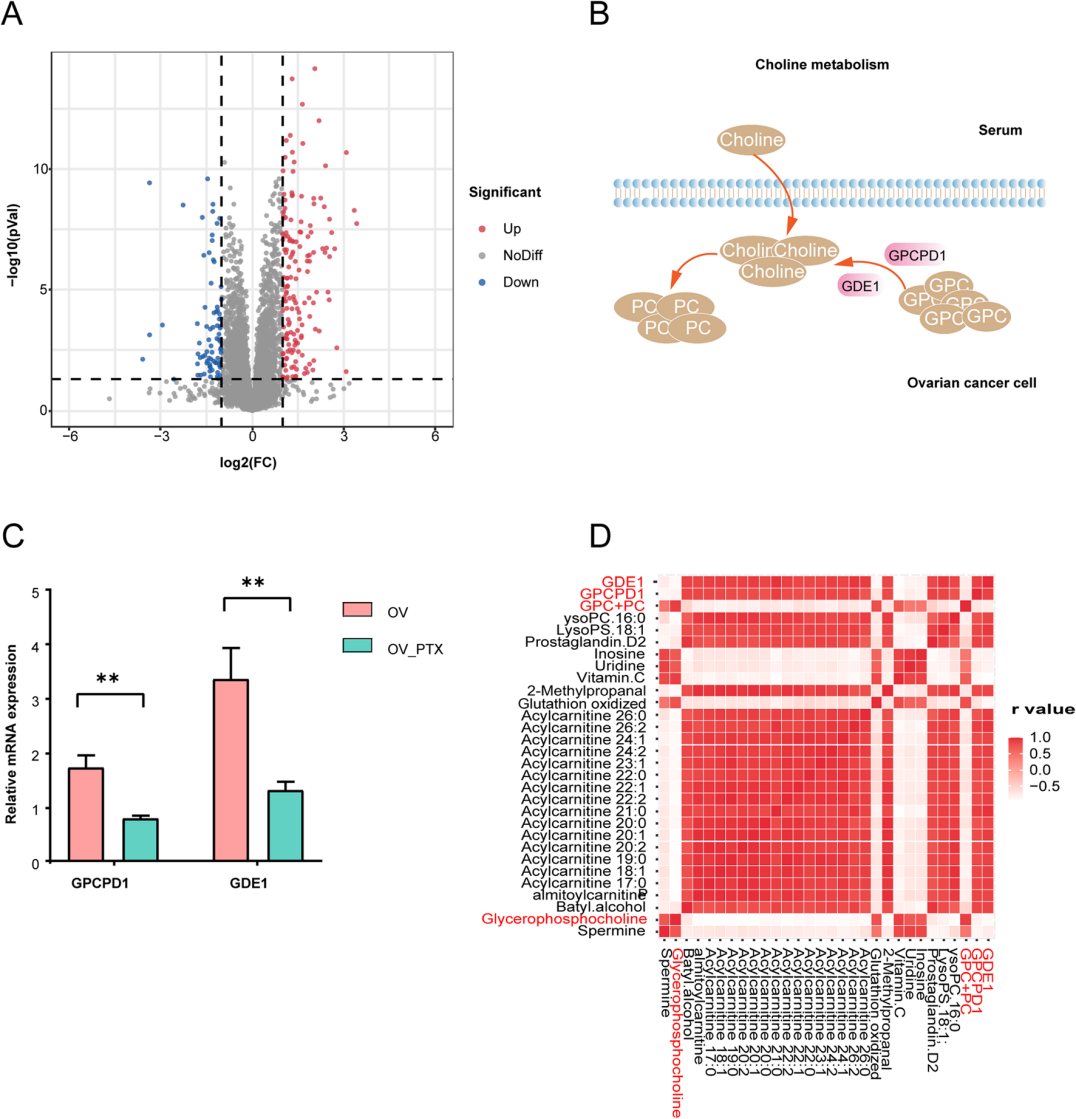
Regulation of GPCPD1 and GDE1 on PTX-resistance in ovarian cancers. Volcano plot showing differential expressed proteins between the OV and OV_PTX groups (**A**). Schematic description of Cho metabolism (**B**). The qRT-PCR showing the expression of GPCPD1 and GDE1 in the OV and OV_PTX groups (**C**). **, *P* < 0.01. Correlation heat map of differential metabolites between GPCPD1 and GDE1 mRNA, in vivo and ex vivo differential metabolites (**D**). *GPCPD1* glycerophosphocholine phosphodiesterase 1, *GDE1* glycerophosphodiester phosphodiesterase 1

According to the major metabolic reprogramming observed in the PTX-resistant EOCs, some essential enzymes might have modulated. Thus we analyzed the proteomics data to identify potential target proteins. A total of 233 differentially expressed proteins, which met fold change ≥ 2 or ≤ 0.5, and *P* < 0.05, were detected between the OV and OV_PTX tumors, without any enzyme of the Cho metabolism (Fig. 6A). GPCPD1 and GDE1 which are responsible for the cleavage of GPC [27] (Fig. 6B) were detectable in the OV tumors, but not in the OV_PTX tumors. Undetectable data in the OV_PTX tumors represented the proteins undetected in over half of the tumors under this experimental methods and conditions. In view of this, we tried to identify GPCPD1and GDE1 since little is known about them in the PTX-resistant EOCs. The expressions of GPCPD1 and GDE1 mRNA were significantly lower in the PTX_OV tumors compared to the OV tumors (both *P* < 0.01) (Fig. 6C).

Next, to address the question that whether gene expression levels of GPCPD1 and GDE1 were related to metabolic outcome, Spearman correlation coefficients were calculated between protein expressions and differential metabolite levels. GPC + PC, the differential metabolite observed on in vivo ^1^H-MRS, correlated negatively with the mRNA levels of GPCPD1 and GDE1, with r = -0.698 (*P* = 0.01) and -0.775 (*P* = 0.003). There were 18 and 26 ex vivo differential metabolites correlated significantly with mRNA levels of GPCPD1 and GDE1 (|r|> 0.8, *P* < 0.05), with a closely negative correlation with GPC (r = -0.841 and -0.912, both *P* < 0.001, respectively) (Fig. 6D). Expressions of enzymes responsible for GPC decomposition reduced in the OV_PTX tumors, leading to the accumulation of GPC.

## Discussion

Our study introduced the application of in vivo ^1^H-MRS in monitoring the PTX resistance of EOC and indicated that glycerophospholipid metabolism might reprogram in the PTX-resistant ovarian cancers. The increased metabolite GPC + PC, namely, Cho, could be detected by in vivo ^1^H-MRS. Major contributions to GPC accumulation may derive from changes in the activities of enzymes (GPCPD1 and GDE1) involved in Cho metabolism, which is part of glycerophospholipid metabolism.

Metabolic regulation does occur in the PTX-resistant tumors. However, few metabolic studies about drug resistance have been reported. Changes in glycerophospholipid metabolism, sphingolipid metabolism and citric acid metabolism have been found in multidrug-resistant colorectal cancer cell lines using metabolomics analysis [28]. In this study, 45 differential metabolites between the OV and OV-PTX tumors were identified by metabolomics analysis, indicating that glucose metabolism, lipid metabolism, amino acid metabolism, energy metabolism, and nucleotide metabolism (purine, pyrimidine metabolism) had been altered. Pathway enrichment analysis further revealed that lipids and lipid-like molecules of lipid metabolism were predominant ex vivo metabolites. Our results were consistent with those reported by Braun et al. In their study, upregulated aerobic glycolysis and activation of pyrimidine synthesis were found in nab-PTX-resistant pancreatic ductal adenocarcinoma cell lines [29].

The PTX-resistant tumors have the altered Cho metabolism. Cho is a major component of glycerophospholipid, and Cho metabolism is an indispensable component of lipid synthesis [5, 30]. Abnormal Cho metabolism has emerged as a hallmark indicator of cancer [31]. Metabolome has revealed that GPC, PC, Cho and Cho complexes are significantly increased in ovarian cancer tissues [32–34]. The tCho level detected by ^1^H-MRS has been evaluated as a diagnostic and prognostic biomarker in cancers [13]. In the differential diagnosis of benign and malignant ovarian tumors, a markedly increased Cho level on ^1^H-MRS is considered to be a characteristic of ovarian cancers [14–17]. Our study showed that there was a significant correlation between ex vivo differential metabolites and GPC + PC on in vivo ^1^H-MRS. GPC was the most significantly upregulated ex vivo metabolite, which indicated that in vivo and ex vivo metabolic characteristics were highly consistent. This observation further suggested that the glycerophospholipids metabolism, especially the Cho metabolism, had changed in the PTX-resistant ovarian cancers. Thus, increased GPC + PC levels observed on ^1^H-MRS in our study which represented tCho, might be the result of Cho metabolism reprogramming in the PTX-resistant ovarian cancers.

The tCho on ^1^H-MRS can monitor PTX resistance of tumors. According to the “Response Evaluation Criteria in Solid Tumors” (RECIST), the tumor size change measured by imaging may require three cycles of chemotherapy to determine therapeutic efficacy [35]. It is reported that patients who showed a greater reduction in tCho than in tumor size are more likely to achieve pathologic complete response [18]. Our ^1^H-MRS findings are accorded with results reported by other researchers that changes of the tCho peak area or height could be used as a biomarker for the therapeutic effect of breast cancer, glioma and liver cancer [18, 22, 36]. A study by Kuo et al. showed that the mean ratios of Cho/lipid were significantly decreased after transcatheter arterial chemoembolization in patients with liver cancer [36]. Another study showed that after treatment with temozolomide for 12 months in patients with low-grade glioma, the tCho was significantly lower than before treatment and after 3 months of treatment [22]. In addition, the Cho/water ratios decreased in accordance with the changes in tumor volume, which suggested that the ratios could reflect the therapeutic effect of temozolomide for glioma [22]. Our in vivo ^1^H-MRS study revealed that GPC + PC (Cho) was significantly higher in the OV_PTX tumors than in OV tumors. Thus, our findings supported that ^1^H-MRS could be used for monitoring PTX-resistant EOCs to allow for early or timely treatment modification.

In addition, our proteomics analysis revealed choline-metabolizing enzymes were low expression in the PTX-resistant EOCs. It is known that GPCPD1 and GDE1 work in a complex enzyme network that regulates Cho metabolism. GPCPD1 cleaves GPC to form glycerol-3-phosphate and Cho [27]. GDE1 catalyzes the hydrolysis of various glycerophosphodiesters (including GPC) and releases sn-glycerol 3-phosphate and the corresponding alcohol [37]. Silencing or upregulating these two enzymes can alter GPC, PC and Cho levels [27]. Homozygous deletion of GDE1 results in a buildup of intracellular GPC that is restored to wild-type levels by reintegrating GDE1 into the genome [38]. In Shen’s transcriptomics analysis of colon adenocarcinoma, the expression of GDE1 is significantly lower than that of normal tissues, indicating abnormal Cho metabolism in malignant tissues [39]. GPCPD1 has been identified as a key enzyme in the Cho and phospholipid metabolism, which is involved in cell proliferation, migration, invasion, adhesion and spreading [27, 40, 41]. It was reported that doxorubicin decreased the expression of GPCPD1, leading to an ex vivo GPC increase in breast cancer cells [42]. Our correlation analysis indicated that the in vivo metabolites and most of the ex vivo metabolites were related to the expressions of GPCPD1 and GDE1. The proteomics analysis showed GPCPD1 and GDE1 expressions were downregulated in the PTX-resistant EOC and confirmed by a qRT-PCR, resulting in a GPC accumulation and an elevated GPC + PC (Cho) peak on ^1^H-MRS. The different expressions of Cho-metabolizing enzymes between the PTX-sensitive and PTX-resistant tumors led to different changes in the GPC, PC and tCho levels, which made GPCPD1 and GDE1 as the potential therapeutic targets.

Our study has some limitations. First, subcutaneous xenograft ovarian cancer models were used in our study, which might differ from orthotopic implantation models due to different biological microenvironments. However, ^1^H-MRS might be difficult to perform in orthotopic ovarian tumors of nude mice due to small tumor sizes and respiratory movements. Second, compared with metabolomics, lipidomics might cover and detect more lipid and lipid-like metabolites, despite the metabolomics results fully explained the ^1^H-MRS findings. Third, Western blot assays were not performed to confirm the protein expression of key enzymes because of limited tumor tissue samples.

In conclusion, our study indicated that the choline metabolic adaptations were associated with the PTX resistance of EOCs. Decreased expressions of GPCPD1 and GDE1 led to increased GPC levels in the PTX-resistant EOCs, which could be observed as the tCho on in vivo ^1^H-MRS. These findings suggested that the tCho on in vivo ^1^H-MRS could be used as an indicator for PTX resistance in EOCs.

## Supplementary Information

**Additional file 1**. Supplementary of metabolomics analysis method.

**Additional file 2**. Supplementary of proteomics analysis method.

**Additional file 3: Figure S1**. PCA scatter plot of the metabolite profile of negative ionization mode between the PTX-sensitive and PTX-resistant tumors. The OV_PTX group was separated from the OV group (A). OPLS-DA analysis showed a good discrimination between the OV and OV_PTX groups, R2 = 0.94, Q2 = -0.49 (B, C).

**Additional file 4: Table S1**. Differential metabolites between OV and OV_PTX groups.

## Data Availability

The datasets used and/or analysed during the current study are available from the corresponding author on reasonable request.

## Abbreviations

AUC: Area under the curve
Ala: Alanine
Asp: Aspartate
Cr: Creatine
EOC: Epithelial ovarian cancer
Ins: Inositol
PTX: Paclitaxel
tCho: Total choline
GDE1: Glycerophosphodiester phosphodiesterase 1
GPC: Glycerophosphocholine
GPCPD1: Glycerophosphocholine phosphodiesterase 1
Lac: Lactate
MM: Macromolecules
Lip: Lipid
NAA: N-acetylaspartate
NAAG: N-acetylaspartylglutamate
OV: OVCAR-3
OV_PTX: Paclitaxel-resistant OVCAR-3
OPLS-DA: Orthogonal partial least squares discriminant analysis
PCA: Principal component analysis
PC: Phosphocholine
PCr: Phosphocreatine
Tau: Taurine
VIP: Variable importance in projection
^1^H-MRS: Proton magnetic resonance spectroscopy
