# Supplementary material for: In vivo detection of dysregulated choline metabolism in paclitaxel-resistant ovarian cancers with proton magnetic resonance spectroscopy
Source: J Transl Med. 2022 Feb 15;20:92. doi: 10.1186/s12967-022-03292-z (PMC8845351; doi:10.1186/s12967-022-03292-z)
Supplement: Supplementary file 1 — Additional file 1. Supplementary of metabolomics analysis method. [file 12967_2022_3292_MOESM1_ESM.doc]

**Supplementary 1**

**Metabolomics analysis**

**Metabolite extraction**

The collected samples were thawed on ice, and metabolites were extracted from 20 µL of each sample using 120 µL of precooled 50% methanol buffer. Then, the mixture of metabolites was vortexed for 1 min, incubated for 10 min at room temperature and stored at -20°C overnight. The mixture was centrifuged at 4,000 g for 20 min; subsequently, the supernatant was transferred to 96-well plates. The samples were stored at -80°C prior to liquid chromatography-mass spectrometry (LC-MS) analysis. Pooled quality control (QC) samples were also prepared by combining 10 µL of each extraction mixture.

**LC-MS analysis**

All samples were analyzed using a Triple time-of-flight (TOF) 5600 Plus high-resolution tandem mass spectrometer (SCIEX, Warrington, UK) operated in both positive and negative ionization modes. Chromatographic separation was performed using an ultraperformance liquid chromatography (UPLC) system (SCIEX, UK). An ACQUITY UPLC T3 column (100 mm * 2.1 mm, 1.8 µm, Waters, UK) was used for reversed-phase separation. For the separation of metabolites, the mobile phase consisted of 0.1% formic acid in water (mobile phase A) and 0.1% formic acid in acetonitrile (mobile phase B). The gradient elution conditions were as follows with a flow rate of 0.4 ml/min: 5% solvent B from 0-0.5 min, 5-100% solvent B from 0.5-7 min, 100% solvent B from 7-8 min, 100-5% solvent B from 8-8.1 min, and 5% solvent B from 8.1-10 min. The column temperature was maintained at 35°C.

The Triple TOF 5600 Plus system was used to detect metabolites eluted from the column. The curtain gas pressure was set at 30 PSI, and the ion source gas 1 and gas 2 pressure was set at 60 PSI. The interface heater temperature was 650°C. For positive ionization mode, the ion spray floating voltage was set at 5 kV, and for negative ionization mode, it was set at ‐4.5 kV. The MS data were acquired in data-independent acquisition (DIA) mode. The TOF mass range was 60-1200 Da. Survey scans were acquired every 150 ms, and as many as 12 product ion scans were collected if the threshold of 100 counts/s was exceeded with a 1+ charge state. The total cycle time was fixed at 0.56 s. Four time bins were summed for each scan at a pulse frequency of 11 kHz by monitoring the 40 GHz multichannel TDC detector with four‐anode/channel detection. Dynamic exclusion was set at 4 s. During the entire acquisition period, the mass accuracy was calibrated every 20 samples. Furthermore, a QC sample was analyzed every 10 samples to evaluate the stability of the LC-MS.

**Metabolomics data processing**

The acquired LC-MS data pretreatment was performed using XCMS software. Raw data files were converted into mzXML format and then processed using the XCMS, CAMERA and metaX toolbox included in R software. Each ion was identified by the comprehensive information of the retention time and m/z. Then, the information was matched to an in-house database and a public database. The open access databases Kyoto Encyclopedia of Genes and Genomes (KEGG, http://www.genome.jp/kegg) and Human Metabolome Database (HMDB, http://www.hmdb.ca), were used to annotate the metabolites by matching the exact molecular mass data (m/z) to those from the database within a threshold of 10 ppm. The peak intensity data was further preprocessed using metaX. Features that were detected in < 50% of QC samples or 80% of test samples were removed, and values for missing peaks were extrapolated with the k‐nearest neighbor algorithm to further improve the data quality. Principal component analysis (PCA) was performed to detect outliers and batch effects using the preprocessed dataset. QC based robust LOESS signal correction was fitted to the QC data with respect to the order of injection to minimize signal intensity drift over time. In addition, the relative standard deviations of the metabolic features were calculated across all QC samples, and those with standard deviations > 30% were removed. The group datasets were normalized before analysis was performed. Data normalization was performed on all samples using the probabilistic quotient normalization algorithm. Then, QC-robust spline batch correction was performed using QC samples. The *P* value analyzed by student t tests which was then adjusted for multiple tests using an FDR (Benjamini-Hochberg) was used for the different metabolite selection. We also conducted the supervised PLS-DA using metaX to variables that discriminant profiling statistical method to identify more specific differences between the groups. The VIP cut-off value of 1.0 was set to select important features.
