# Supplementary material for: In vivo detection of dysregulated choline metabolism in paclitaxel-resistant ovarian cancers with proton magnetic resonance spectroscopy
Source: J Transl Med. 2022 Feb 15;20:92. doi: 10.1186/s12967-022-03292-z (PMC8845351; doi:10.1186/s12967-022-03292-z)
Supplement: Supplementary file 2 — Additional file 2. Supplementary of proteomics analysis method. [file 12967_2022_3292_MOESM2_ESM.doc]

**Supplementary 2**

**Proteomics analysis**

**Protein extraction**

Samples were lysed in sodium dodecyl sulfate (SDS) buffer and homogenized by an MP Fastprep-24 automated homogenizer. After the homogenate was sonicated, boiled and centrifuged, the supernatant was filtered. Iodoacetamide (100 mM) was added to block reduced cysteine residues and the samples were incubated for 30 min in the dark. Finally, the protein suspensions were digested with trypsin (Promega, Madison, WI, USA) overnight at 37°C, and the resulting peptides were collected as a filtrate.

**DIA analysis**

Pooled peptides from all samples were fractionated by reversed-phase chromatography using an Agilent 1260 infinity II HPLC (SCIEX, Framingham, MA, USA). The peptide mixture was diluted with buffer A (5% acetonitrile) and loaded onto a chromatographic column (XBridge Peptide BEH C18 Column, 130 A, 5 µm, 4.6 mm × 100 mm). The peptides were eluted at a flow rate of 1 mL/min with a gradient of 0-7% buffer B (85% acetonitrile) for 5 min, 7-40% buffer B from 5-40 min, 40-100% buffer B from 45-50 min, and 100% buffer B from 50-65 min. Fractions were collected every minute from 5-50 min. The collected fractions were dried down via vacuum centrifugation at 45°C.

The peptides were redissolved in solvent A (A: 0.1% formic acid in water) and analyzed by on-line nanospray LC-MS/MS on an Orbitrap Exploris 480 coupled to an EASY-nLC system (Thermo Fisher Scientific, MA, USA). A 1 µg peptide sample was loaded on an analytical column (Nano Technology Column, 18 cm C18 column, with 1.9 μm C18 Resin, Catalog Number: 26350-3) and separated within a 120-min gradient from 2% to 35% B (B: 0.1% formic acid, 80% acetonitrile). The column flow rate was maintained at 300 nL/min. An electrospray voltage of 2056 V versus the inlet of the mass spectrometer was used.

**Proteomics data processing**

Raw data of data-dependent acquisition (DDA) were processed and analyzed by Spectronaut 14.6 (Biognosys AG, Switzerland) with default settings to generate an initial target list. Spectronaut was set up to search the database of Uniprot_MusMusculus_17027_20200226 (http://www.UniProt.org) assuming trypsin was the digestion enzyme. Carbamidomethyl was specified as the fixed modification. Oxidation and acetyl were specified as the variable modifications. Q value (FDR) cutoff on precursor and protein level was applied 1%.

Raw data of DIA were processed and analyzed by Spectronaut 14.6 (Biognosys AG, Switzerland) with default settings. The retention time prediction type was set to dynamic iRT. Data extraction was determined by Spectronaut X based on extensive mass calibration. Spectronaut 14.6 dynamically determined the ideal extraction window depending on iRT calibration and gradient stability. A Q value (false discovery rate, FDR) cutoff of 1% was applied to the precursor and protein level. All selected precursors passing the filters were used for quantification. MS2 interference removed all interfering fragment ions except for the 3 least interfering ions. The average top 3 filtered peptides that passed the 1% Q value cutoff were used to calculate the major group quantities. Significantly enriched proteins were selected using a two-sided t-test analysis with a 5% FDR.
