# Supplementary figures and images for: In vivo detection of dysregulated choline metabolism in paclitaxel-resistant ovarian cancers with proton magnetic resonance spectroscopy
Source: J Transl Med. 2022 Feb 15;20:92. doi: 10.1186/s12967-022-03292-z (PMC8845351; doi:10.1186/s12967-022-03292-z)

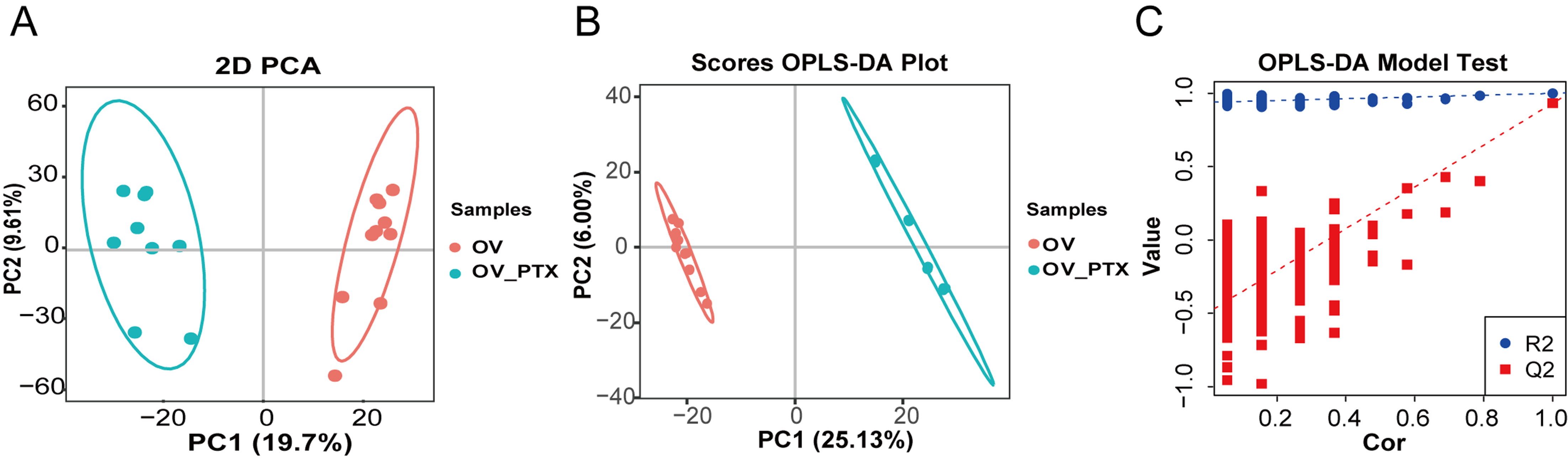

Supplement: Supplementary file 3 — Additional file 3: Figure S1. PCA scatter plot of the metabolite profile of negative ionization mode between the PTX-sensitive and PTX-resistant tumors. The OV_PTX group was separated from the OV group (A). OPLS-DA analysis showed a good discrimination between the OV and OV_PTX groups, R2 = 0.94, Q2 = -0.49 (B, C). [file 12967_2022_3292_MOESM3_ESM.tif]
