# Supplementary material for: In vivo detection of dysregulated choline metabolism in paclitaxel-resistant ovarian cancers with proton magnetic resonance spectroscopy
Source: J Transl Med. 2022 Feb 15;20:92. doi: 10.1186/s12967-022-03292-z (PMC8845351; doi:10.1186/s12967-022-03292-z)
Supplement: Supplementary file 4 — Additional file 4: Table S1. Differential metabolites between OV and OV_PTX groups. [file 12967_2022_3292_MOESM4_ESM.doc]

Supplementary Table 1 Differential metabolites between OV and OV_PTX groups

| Metabolites | Metabolite class | Ratio | VIP |
| --- | --- | --- | --- |
| Spermidine | Organic nitrogen compounds | 0.15 | 3.93 |
| Spermine | Organic nitrogen compounds | 0.08 | 4.19 |
| 1-Methyl-6-phenyl-1H-imidazo[4,5-b]pyridin-2-amine | Organoheterocyclic compounds | 0.21 | 3.68 |
| Glycerophosphocholine | Lipids and lipid- like molecules | 0.42 | 3.13 |
| Guanosine | Nucleosides, nucleotides, and analogues | 2.26 | 2.07 |
| Batyl alcohol | Lipids and lipid-like molecules | 2.44 | 2.53 |
| Acylcarnitine 14:0 | Lipids and lipid-like molecules | 2.91 | 3.12 |
| Acylcarnitine 16:1 | Lipids and lipid-like molecules | 2.32 | 2.08 |
| Palmitoylcarnitine | Lipids and lipid-like molecules | 3.36 | 2.84 |
| Acylcarnitine 17:0 | Lipids and lipid-like molecules | 2.71 | 2.85 |
| Acylcarnitine 18:1 | Lipids and lipid-like molecules | 2.41 | 2.23 |
| Acylcarnitine 19:0 | Lipids and lipid-like molecules | 4.45 | 3.20 |
| Acylcarnitine 20:2 | Lipids and lipid-like molecules | 2.72 | 2.68 |
| Acylcarnitine 20:1 | Lipids and lipid-like molecules | 2.7 | 2.34 |
| Acylcarnitine 20:0 | Lipids and lipid-like molecules | 4.05 | 2.47 |
| Acylcarnitine 21:0 | Lipids and lipid-like molecules | 4.18 | 2.69 |
| Acylcarnitine 22:2 | Lipids and lipid-like molecules | 3.2 | 2.54 |
| Acylcarnitine 22:1 | Lipids and lipid-like molecules | 2.69 | 1.93 |
| Acylcarnitine 22:0 | Lipids and lipid-like molecules | 3.21 | 2.08 |
| Acylcarnitine 23:1 | Lipids and lipid-like molecules | 3.71 | 2.72 |
| Acylcarnitine 23:0 | Lipids and lipid-like molecules | 3.48 | 2.43 |
| Acylcarnitine 24:2 | Lipids and lipid-like molecules | 2.79 | 2.10 |
| Acylcarnitine 24:1 | Lipids and lipid-like molecules | 2.24 | 1.54 |
| Acylcarnitine 24:0 | Lipids and lipid-like molecules | 2.38 | 1.85 |
| Taurocholate | Lipids and lipid-like molecules | 0.18 | 4.04 |
| Acylcarnitine 26:2 | Lipids and lipid-like molecules | 2.26 | 1.56 |
| Acylcarnitine 26:1 | Lipids and lipid-like molecules | 2.15 | 1.81 |
| Acylcarnitine 26:0 | Lipids and lipid-like molecules | 2.64 | 2.48 |
| Glutathione, oxidized | Organic acids and derivatives | 0.41 | 2.00 |
| 5-Aminopentanoic acid | Organic acids and derivatives | 0.43 | 2.37 |
| 2-Methylpropanal | Organooxygen compounds | 2.51 | 2.18 |
| Xanthine | Organoheterocyclic compounds | 0.38 | 2.35 |
| L-Cysteine Sulfinic acid | Organic acids and derivatives | 0.48 | 2.23 |
| N-Acetyl-L-aspartic acid | Organic acids and derivatives | 0.35 | 1.99 |
| Vitamin C | Organoheterocyclic compounds | 0.13 | 3.86 |
| Citric acid | Organic acids and derivatives | 2.16 | 1.97 |
| Uridine | Nucleosides, nucleotides, and analogues | 0.46 | 2.43 |
| Inosine | Nucleosides, nucleotides, and analogues | 0.48 | 2.54 |
| N-Acetylneuraminic acid | Organic oxygen compounds | 0.44 | 2.46 |
| Prostaglandin D2 | Lipids and lipid-like molecules | 2.09 | 2.28 |
| Docosahexaenoic acid | Lipids and lipid-like molecules | 2.19 | 1.68 |
| LysoPE 18:2 | Lipids and lipid-like molecules | 2.37 | 2.47 |
| LysoPE 20:3 | Lipids and lipid-like molecules | 2.06 | 2.16 |
| LysoPS 18:1; LysoPS 18:1 | Lipids and lipid-like molecules | 2.04 | 2.31 |
| LysoPC 16:0 | Lipids and lipid-like molecules | 3.19 | 2.70 |

Ratio: OV group vs OV_PTX group; VIP: variable importance in projection in orthogonal partial least squares discriminant analysis.
